# Rapidly-Exploring Adaptive Sampling Tree*: A Sample-Based Path-Planning Algorithm for Unmanned Marine Vehicles Information Gathering in Variable Ocean Environments

**DOI:** 10.3390/s20092515

**Published:** 2020-04-29

**Authors:** Chengke Xiong, Hexiong Zhou, Di Lu, Zheng Zeng, Lian Lian, Caoyang Yu

**Affiliations:** 1 School of Oceanography, Shanghai Jiao Tong University, Shanghai 200240, China; xiongchengke@sjtu.edu.cn (C.X.); zhou_hexiong@sjtu.edu.cn (H.Z.); llltom@sjtu.edu.cn (D.L.); llian@sjtu.edu.cn (L.L.); yucaoyang@sjtu.edu.cn (C.Y.); 2 State Key Laboratory of Ocean Engineering, Shanghai Jiao Tong University, Shanghai 200240, China; 3 Qingdao Collaborative Innovation Center of Marine Science and Technology, Qingdao 266100, China

**Keywords:** path planning, unmanned marine vehicles, adaptive ocean sampling, rapidly-exploring adaptive sampling tree star

## Abstract

This research presents a novel sample-based path planning algorithm for adaptive sampling. The goal is to find a near-optimal path for unmanned marine vehicles (UMVs) that maximizes information gathering over a scientific interest area, while satisfying constraints on collision avoidance and pre-specified mission time. The proposed rapidly-exploring adaptive sampling tree star (RAST*) algorithm combines inspirations from rapidly-exploring random tree star (RRT*) with a tournament selection method and informative heuristics to achieve efficient searching of informative data in continuous space. Results of numerical experiments and proof-of-concept field experiments demonstrate the effectiveness and superiority of the proposed RAST* over rapidly-exploring random sampling tree star (RRST*), rapidly-exploring adaptive sampling tree (RAST), and particle swarm optimization (PSO).

## 1. Introduction

The ocean is a complex dynamical system. To understand ocean dynamics and increase accuracy of ocean models, measurements need to be taken with high spatio-temporal resolution according to the ocean phenomena under investigation [1,2,3,4].

Unmanned marine vehicles (UMVs), such as unmanned surface vehicles (USVs), gliders, and autonomous underwater vehicles (AUVs), equipped with sensors, have been widely employed to collect informative data of all kinds over scientific interest areas, for example, salinity, temperature, and chlorophyll content of the ocean, for applications of ecosystem monitoring, pollution management, marine resources exploration, and utilization [5,6,7,8,9,10]. However, a key challenge arises in that the vehicles are subject to limited energy resource or mission time, which limits data collection of the vehicle in one mission [11,12,13,14]. Therefore, it is of vital importance that the UMV can automatically plan an informative trajectory in variable ocean environments while satisfying constraints on collision avoidance, limited energy, and pre-specified mission time [15,16,17].

A variety of informative path planning methods have been proposed for adaptive sampling in an ocean environment. Mixed integer linear programming (MILP) is applied to find the path of AUV for adaptive sampling in the region of the greatest uncertainty [18]. The branch and bound (BNB) method is proposed for informative path planning to maximize the average reduction in variance of the estimated field [19]. MILP and BNB methods are easy to be implemented but are limited to discrete domains and often scale poorly in the size of the ocean environment. Rapidly exploring information gathering (RIG) algorithms are proposed to find a trajectory that maximizes an information quality metric within a pre-specified budget constraint [20]. While RIG algorithms can achieve efficient information gathering in continuous space with motion constraints, informative heuristics is not applied to guide the robot to higher information quality areas. Ma et al.[21,22] present an informative path planning method to maximize information gathering through sampling the environment; however, the method only provides guarantees on suboptimal solutions. A multi-objective particle swarm optimization (MOPSO) with a fuzzy comprehensive evaluation (FCE) method is proposed to formulate AUVs adaptive sampling problem while considering a spatiotemporal ocean environment and energy constraints [23]. The method can plan an AUV path in continuous space but requires heavy computational burden in a high dimensional search space.

Sample-based algorithms, such as rapidly-exploring random tree (RRT) [24], probabilistic roadmap (PRM) [25], and variants of them, with the advantage of generating a collision-free path quickly in continuous space, have been studied extensively in the past years. Rapidly-exploring random tree star (RRT*) [26], a variant of standard RRT, is guaranteed asymptotic optimality and has been widely used for solving the path planning problem [27,28,29,30,31]. Therefore, RRT* can be an alternative choice to be employed in the informative path planning problem for adaptive sampling. However, when it comes to maximum information gathering, standard RRT* is inefficient because the heuristics of distance in the algorithm structure gives no positive guidance for UMVs to collect informative data in regions of high scientific interest. Therefore, this research presents a variant of RRT*, referred to as rapidly-exploring adaptive sampling tree star (RAST*), that combines inspirations from RRT* with the tournament selection method and informative heuristics to achieve efficient searching of informative data in continuous space.

The novelty of RAST* includes: (1) utilizing the tournament selection method in replacement of random sample, so that more new branches will fall in higher scientific interest areas, which can result in finding optimal solutions quickly and saving computation time; (2) modifying the heuristic procedure from distance to information gathering per hour in order to grow branches that can gain more information with less traveling time. To our knowledge, this is the first work to integrate a tournament selection method, informative heuristic function, and sample-based algorithm into a unified path planner.

To evaluate the performance of the proposed RAST* path planner, different scenarios are designed to compare the proposed RAST* path planner with rapidly-exploring random sampling tree star (RRST*), rapidly-exploring adaptive sampling tree (RAST), and particle swarm optimization (PSO). Additionally, proof-of-concept field experiments are performed in Lake Zhiyuan to assess the superiority and effectiveness of the proposed RAST* path planner.

The remainder of this research is organized as follows. Section 2 gives a detailed description of the mathematical model of the path planning problem for adaptive sampling. Section 3 outlines four path planning methods. Section 4 presents numerical experiments and results of different scenarios. Section 5 presents field experiments and results. Section 6 draws the conclusions and discusses future works.

## 2. Problem Formulation

This section formulates the path planning problem for adaptive sampling using a UMV. The objective is to find optimal path $P^{*}$ among all feasible paths P that maximizes information gathering over an ocean area with features of scientific interest A and ocean currents $V_{c}$ while considering constraints of obstacles O and pre-specified mission time T.

Let A be the utility map generated by the needs of marine scientists and assumed to be known a priori. UMVs equipped with a CTD sensor or chlorophyll fluorometer can be used to measure the scientific interest areas. The utility map, a two-dimensional (2D) matrix with probability value in [0,1], is a combination of features of scientific interest, such as salinity, chlorophyll fluorescence, dissolved oxygen, turbidity, etc. [32] The areas with a higher probability value of interest in the utility map are where scientists desire the UMVs to detect and collect relevant informative data. Ocean currents data $V_{c}$ are obtained from the National Oceanic and Atmospheric Administration (NOAA). The utility map A is generated based on sea temperature data from NOAA in this research. For more details, see: https://www.ncdc.noaa.gov/.

In practice, the path planning problem for UMV adaptive sampling operation is generally solved for long-term missions with durations of several days and trajectory lengths of hundreds of kilometers. The effect of vehicle dynamics is considered negligible and the vehicle is regarded as a point for the scale of this planning problem.

Considering that a UMV V is deployed to travel with velocity V from its initial position $P_{1}$ = [$x_{1}$, $y_{1}$] to detect and collect ocean data, the potential path is represented by a sequence of discrete points $P=\{P_{1},P_{2},...,P_{h}\}$, where *h* is the number of discrete points along the path and $P_{h}$ is the final position for the whole mission.

The path planning problem for adaptive sampling can be expressed as

(1) $$\begin{array}{cc} \; & P^{*}=\underset{P\in P}{argmax}\;IG\left(V,V,V_{c},A,O,T\right)\\ s.t.\; & \dot{V}=0,\\ \; & \forall i\in \left\{1,2,...,h\right\}\;P_{i}\notin O\\ \; & T<T \end{array}$$

where IG is the fitness function representing the information gathering for the whole mission.

### 2.1. Fitness Function Evaluation

Since informative data is associated with probability value of interest in the utility map, the goal of this research can be equivalently transformed to maximize the total probability value of interest along a potential path. Therefore, the fitness function can be formulated as the integral of probability value of interest along a potential path.

(2) $$\begin{array}{c} IG=\int_{1}^{h}A\left(P_{i}\right)\cdot \varepsilon_{i}\cdot di \end{array}$$

(3) $$\varepsilon_{i}=\left\{\begin{array}{cc} 1, & if\;distance\left(P_{i},P_{j}\right)>d_{min},\forall j=1,...,i-1\\ 0, & else \end{array}\right.$$

where $A(P_{i})$ is the probability value surrounding sample point $P_{i}$, $\varepsilon_{i}$ is a parameter that evaluates whether the current position of the UMV in ocean environments has been sampled, $d_{min}$ is the sensor range. If the distance between the current position $P_{i}$ of the UMV and the sampled waypoints is larger than $d_{min}$, the probability value $A(P_{i})$ will be recorded, in case of repeated sampling.

### 2.2. Traveling Time Calculation

The UMV is assumed to keep constant thrust power and, equivalently, constant speed *V* during the whole mission. Influenced by ocean currents $V_{c}$, the water-referenced velocity of the UMV at a sample point in position (x,y) at time *t* can be obtained from Equation (4).

(4) $$\begin{array}{cc} \; & u_{t}=V_{cu}^{t-xy}+V\cos\phi_{t}\\ \; & v_{t}=V_{cv}^{t-xy}+V\sin\phi_{t}\\ \; & V_{t}=\sqrt{{u_{t}}^{2}+{v_{t}}^{2}} \end{array}$$

where $V_{cu}^{t-xy}$ and $V_{cv}^{t-xy}$ are the velocity components of ocean currents $V_{c}$ in position (x,y) at time *t*, $\phi_{t}$ are the heading angle of the UMV at time *t*.

Hence, traveling time *T* for one potential path can be calculated by the sum of time required for each discrete line segment.

(5) $$\begin{array}{c} T=\sum_{i=1}^{i=h-1}\frac{|P_{i+1}-P_{i}|}{V_{t}} \end{array}$$

where $V_{t}$ should be recalculated for each discrete line segment.

## 3. Methods

This section presents four path planning methods for adaptive sampling. The key idea is to generate the trajectory of UMV that maximizes information gathering over an ocean area with features of scientific interest in variable ocean environments. In addition, constraints of obstacles in environments and a pre-specified mission time of UMV is taken into consideration. Meanwhile, configuration space is pre-defined by marine scientists based on their interests. It can be any type of feature field, such as error variances, represented by uncertainty, or physical features of interest (temperature, salinity, eddies, etc.). A more generic setting is the higher value of a certain position in the feature field, indicating that it is generally worthwhile to send the UMV to take measurements. For close-to-reality test, the data for the features used in this study were obtained from the NOAA, in which irregularly shaped islands are regarded as obstacles. Normalized sea temperature data are utilized as the utility map.

### 3.1. RAST*

RAST* builds a tree structure by connecting a set of nodes sampled from the configuration space and returns an optimal collision-free path. The pseudocode of RAST* is shown in Algorithm 1.

RAST* starts with initial node $q_{init}$ in Vertex and empty sets of *E* and $V_{invalid}$. In the main loop, instead of using the random sample procedure, the tournament selection method in Algorithm 2 is newly applied to elect $q_{ts}$ among M candidate nodes that are randomly sampled from the configuration space A. (Line 3) Then, the HeuristicNode function in Algorithm 3, with ocean currents taking into consideration when calculating the traveling time, is newly developed to find the node $q_{maximum}$ among all existing nodes in Vertex such that the vector line from $q_{maximum}$ to $q_{ts}$ is with the maximum information gathering per hour. If ocean currents are greater than the maximum speed of the UMV, the algorithm will not generate branches towards this direction and will choose other nodes to extend branches. (Line 4) After that, $q_{new}$ is generated by extending a step with step size δ from $q_{maximum}$ to $q_{ts}$ based on Algorithm 4. (Line 5) If the line segment between $q_{maximum}$ and $q_{new}$ is collision free, which is checked by Algorithm 5 (Line 6), Algorithm 6 will be applied to find out a neighbor nodes set $Q_{m}$. If the distance from any nodes in Vertex to $q_{new}$ is less than radius *r*, the node will be included in the neighbor nodes set $Q_{m}$. Then, Algorithm 7 is executed to find the node $q_{max}$ that can make the path, from $q_{init}$ passing connected nodes until reaching $q_{max}$ then to $q_{new}$, with the maximum information gathering per hour. (Line 7–15) Searching is performed by the newly developed HeuristicPath function. If the line segment between $q_{max}$ and $q_{new}$ is collision free (Line 16), a new node $q_{new}$ will be added in Vertex, a new branch $\left(q_{max},q_{new}\right)$ will be added in *E*, $q_{max}$ will be the parent node of $q_{new}$, the traveling time and information gathering from $q_{init}$ to $q_{new}$ will be calculated. (Line 17–21) Additionally, if traveling time from $q_{init}$ to $q_{new}$ exceeds the pre-specified mission time T, $q_{new}$ will be marked as the invalid nodes $V_{invalid}$. (Line 22–24) At the end of the main loop, the ratio $P_{r}$ is calculated as the number of invalid nodes to the number of all existing nodes. (Line 27) The operator terminates the optimization when the first time that *P* exceeds a predefined maximum ratio value $P_{r}$ and outputs the optimal path together with correlated information gathering.

RAST* differentiates itself from the other variants of RRT* in that tournament sample procedure and informative heuristic function are newly developed for path optimization in the algorithm structure. The arrangement of the tournament sample makes RAST* attempt to grow branches towards high scientific interest areas. The reason why the informative heuristic function is denoted as information gathering per hour is that it is beneficial to gain more information with less traveling time. To this end, these two contributions will result in generating the optimized path of UMV with more information gathering for an adaptive sampling mission.
**Algorithm 1** RAST***Input**:
Utility map A, ocean currents data $V_{c}$, velocity of UMV V, initial position of UMV $q_{init}$, obstacles O, pre-specified mission time T, tournament parameter M, step size δ, radius *r*, predefined maximum ratio value $P_{r}$. 1:Vertex←{$q_{init}$}; Edge←*⌀*; $V_{invalid}$←*⌀*; Graph=(Vertex,Edge); 2:**while**$P<P_{r}$**do** 3:    $q_{ts}$←TournamentSample(A, M); 4:    $q_{maximum}$←HeuristicNode($q_{ts}$,Vertex); 5:    $q_{new}$←Steer($q_{maximum}$,$q_{ts}$,δ); 6:    **if** CollisionFree($q_{maximum}$,$q_{new}$) **then** 7:        $Q_{m}\leftarrow Near\left(Vertex,q_{new},r\right)$; 8:        $q_{max}$←$q_{maximum}$; 9:        $c_{max}$←HeuristicPath($q_{new}$,$q_{maximum}$,$q_{init}$); 10:        **for** each $q_{m}\in Q_{m}$ **do** 11:           **if** HeuristicPath($q_{new}$,$q_{m}$,$q_{init}$)>$c_{max}$ **then** 12:               $c_{max}$←HeuristicPath($q_{new}$,$q_{m}$,$q_{init}$); 13:               $q_{max}$←$q_{m}$; 14:           **end if** 15:        **end for** 16:        **if** CollisionFree($q_{max}$,$q_{new}$) **then** 17:           $Vertex\leftarrow Vertex\cup \{q_{new}\}$;  18:           $Edge\leftarrow Edge\cup \{\left(q_{max},q_{new}\right)\}$; 19:           $q_{new}.Parent\leftarrow q_{max}$;  20:           $q_{new}.T\leftarrow Time\left(q_{new},Vertex,Edge\right)$;  21:           $q_{new}.IG\leftarrow FitnessFun\left(q_{new},Vertex,Edge\right)$;  22:           **if** $q_{new}.T>T$ **then** 23:               $V_{invalid}\leftarrow V_{invalid}\cup \left\{q_{new}\right\}$;  24:           **end if** 25:        **end if** 26:    **end if** 27:    $P=Ratio(V_{invalid},Vertex)$; 28:**end while** 29:**return**Graph=(Vertex,Edge); **Output**:
The best fitness value and its correlated paths as the optimal solutions.

**Algorithm 2** Tournament Sample
1:**function**TournamentSample(A,M) 2:    Select M random points {$q_{1},q_{2},...,q_{M}$} from the utility map A; 3:    $q_{ts}\leftarrow q_{1}$; 4:    **for** i=2 to M **do**  5:        **if** $A\left(q_{i}\right)>A\left(q_{ts}\right)$ **then** 6:           $q_{ts}\leftarrow q_{i}$; 7:        **end if** 8:    **end for** 9:    **return** $q_{ts}$ 10:
**end function**



**Algorithm 3** Heuristic Node
1:**function**HeuristicNode($q_{ts}$,Vertex) 2:    vn = Length(Vertex); %Find out how many nodes exist in Vertex  3:    mr = 0;  4:    **for** i=1 to vn **do**  5:        $IG_{i}=Information\left(q_{i},q_{new}\right)$; %The information can be gathered from $q_{i}$ to $q_{new}$  6:        $T_{i}=Time\left(q_{i},q_{new}\right);$  7:        $rate_{i}=IG_{i}/T_{i};$ 8:        **if** $rate_{i}>mr$ **then**  9:           $q_{maximum}\leftarrow q_{i}$. 10:           $mr=rate_{i}$; 11:        **end if** 12:    **end for** 13:    **return** $q_{maximum}$  14:
**end function**



**Algorithm 4** Steer
1:**function**Steer($q_{maximum}$,$q_{ts}$,δ) 2:    D = distance($q_{maximum}$,$q_{ts}$); 3:    **if** D > δ **then** 4:        $q_{new}=q_{ts}+\left(q_{maximum}-q_{ts}\right)*\delta /D$  5:    **else** 6:        $q_{new}\leftarrow q_{maximum}$ 7:    **end if** 8:    **return** $q_{new}$  9:
**end function**



**Algorithm 5** Collision Free
1:**function**CollisionFree($q_{i},q_{j}$) 2:    **if** Line segment from $q_{i}$ to $q_{j}$ doesn’t collide with obstacles. **then** 3:        **return** 1  4:    **else** 5:        **return** 0  6:    **end if** 7:
**end function**



**Algorithm 6** Near
1:**function**Near($Vertex,q_{new},r$) 2:    $Q_{m}\leftarrow ⌀$ 3:    Find out how many nodes exist in Vertex and record the number as *v*. 4:    **for** i=1 to *v* **do**. 5:        **if** $distance(q_{i},q_{new})<r$ **then** 6:           $Q_{m}\leftarrow Q_{m}\cup q_{i}$ 7:        **end if** 8:    **end for** 9:    **return** $Q_{m}$  10:
**end function**



**Algorithm 7** Heuristic Path
1:**function**HeuristicPath($q_{new}$,$q_{m}$,$q_{init}$) 2:    $P=Connection(q_{new},q_{m},q_{init})$; %Connect branches from $\left(q_{new},q_{m}\right)$ back to the initial position $q_{init}$ 3:    IG=FitnessFun(P); 4:    T=Time(P); 5:    $c_{max}=IG/T$; 6:    **return** $c_{max}$  7:
**end function**



### 3.2. RRST*

RRST* is a variant of RRT*, and the idea behind this variant is similar to RAST* except that RRST* retains the random sample procedure of RRT* in Line 3. As for RRST*, Line 3 is replaced as $q_{ts}\leftarrow RandomSample\left(A\right)$; and the rest of the procedures of RRST* are the same as RAST*, shown in Algorithm 8. The RRST* randomly selects a point $q_{ts}$ from the configuration space to extend its branch. Hence, the main advantage of RRST* is that it samples the configuration space with more randomness. Due to the randomness of RRST*, RRST* may need more iterations to find the optimized solution, and result in requiring more computational time than RAST*.
**Algorithm 8** RRST***Input**:
Utility map A, ocean currents data $V_{c}$, velocity of UMV V, initial position of UMV $q_{init}$, obstacles O, pre-specified mission time T, step size δ, radius *r*, predefined maximum ratio value $P_{r}$. 1:Vertex←{$q_{init}$}; Edge←*⌀*; $V_{invalid}$←*⌀*; Graph=(Vertex,Edge); 2:**while**$P<P_{r}$**do** 3:    $q_{ts}$←RandomSample(A); 4:    $q_{maximum}$←HeuristicNode($q_{ts}$,Vertex); 5:    $q_{new}$←Steer($q_{maximum}$,$q_{ts}$,δ); 6:    **if** CollisionFree($q_{maximum}$, $q_{new}$) **then** 7:        $Q_{m}\leftarrow Near\left(Vertex,q_{new},r\right)$; 8:        $q_{max}$←$q_{maximum}$; 9:        $c_{max}$←HeuristicPath($q_{new}$, $q_{maximum}$,$q_{init}$); 10:        **for** each $q_{m}\in Q_{m}$ **do** 11:           **if** HeuristicPath($q_{new}$, $q_{m}$, $q_{init}$)>$c_{max}$ **then** 12:               $c_{max}$←HeuristicPath($q_{new}$,$q_{m}$,$q_{init}$); 13:               $q_{max}$←$q_{m}$; 14:           **end if** 15:        **end for** 16:        **if** CollisionFree($q_{max}$,$q_{new}$) **then** 17:           $Vertex\leftarrow Vertex\cup \{q_{new}\}$;  18:           $Edge\leftarrow Edge\cup \{\left(q_{max},q_{new}\right)\}$; 19:           $q_{new}.Parent\leftarrow q_{max}$;  20:           $q_{new}.T\leftarrow Time\left(q_{new},Vertex,Edge\right)$;  21:           $q_{new}.IG\leftarrow FitnessFun\left(q_{new},Vertex,Edge\right)$;  22:           **if** $q_{new}.T>T$ **then** 23:               $V_{invalid}\leftarrow V_{invalid}\cup \left\{q_{new}\right\}$;  24:           **end if** 25:        **end if** 26:    **end if** 27:    $P=Ratio(V_{invalid},Vertex)$; 28:**end while** 29:**return**Graph=(Vertex,Edge); **Output**:
The best fitness value and its correlated paths as the optimal solutions.

### 3.3. RAST

RAST is a variant of RRT. The main loop of the RAST follows the general structure outlined in Algorithm 9. As can be seen from Algorithms 1 and 9, the difference between these two algorithms lies in that RAST lacks the procedures of connecting more “informative” branches in Line 7–15 in Algorithm 1. Even though, this approach can guide the branches to grow towards high scientific interest areas. However, similar to RRT, RAST is not asymptotically optimal.
**Algorithm 9** RAST**Input**:
Utility map A, ocean currents data $V_{c}$, velocity of UMV V, initial position of UMV $q_{init}$, obstacles O, pre-specified mission time T, tournament parameter M, step size δ, predefined maximum ratio value $P_{r}$. 1:Vertex←{$q_{init}$}; Edge←*⌀*; $V_{invalid}$←*⌀*; Graph=(Vertex,Edge); 2:**while**$P<P_{r}$**do** 3:    $q_{ts}$←RandomSample(A, M); 4:    $q_{maximum}$←HeuristicNode($q_{ts}$,Vertex); 5:    $q_{new}$←Steer($q_{maximum}$,$q_{ts}$,δ); 6:    **if** CollisionFree($q_{max}$,$q_{new}$) **then** 7:        $Vertex\leftarrow Vertex\cup \{q_{new}\}$;  8:        $Edge\leftarrow Edge\cup \{\left(q_{max},q_{new}\right)\}$; 9:        $q_{new}.Parent\leftarrow q_{max}$;  10:        $q_{new}.T\leftarrow Time\left(q_{new},Vertex,Edge\right)$;  11:        $q_{new}.IG\leftarrow FitnessFun\left(q_{new},Vertex,Edge\right)$;  12:        **if** $q_{new}.T>T$ **then** 13:           $V_{invalid}\leftarrow V_{invalid}\cup \left\{q_{new}\right\}$;  14:        **end if** 15:    **end if** 16:    $P=Ratio(V_{invalid},Vertex)$; 17:**end while** 18:**return**Graph=(Vertex,Edge); **Output**:
The best fitness value and its correlated paths as the optimal solutions.

### 3.4. PSO

PSO is a population-based stochastic optimization algorithm discovered through simulation of the social behavior of a bird flock [33]. PSO is possessed with the advantages of easy implementation and sufficient solution diversity. Therefore, PSO is compared with the three aforementioned methods. The overview of the PSO path planner can be found in [34,35], and the pseudo code of PSO is shown in Algorithm 10.

### 3.5. Computational Complexity

The time complexity for RRT is discussed in detail in [26] and given as *O*(*n*log*n*), where *n* denotes the total number of iterations. Time complexity is defined as the amount of time that is required by the algorithm to find the solution. RAST*, RRST*, and RAST are inspired by RRT, the additional steps of intelligent sampling procedure that have been introduced in these three algorithms have complexities that are insignificant enough to have an effect on the complexity of the algorithm. Hence, these three algorithms have almost the same time complexity as that of RRT, but the value of *n* can be significantly reduced in terms of RAST*. RAST* is able to generate better solutions because the newly generated nodes are concentrated towards high scientific interest regions.
**Algorithm 10** PSO**Input**:
Utility map A, ocean currents data $V_{c}$, velocity of UMV V, initial position of UMV $P_{1}$, obstacles O, pre-specified mission time T, parameter c1, c2, *w* and $w_{damp}$, population size *K*, maximum number of iterations Iter and number of control points *n*. 1:Initialize the position and velocity of each particle. 2:**for***i* = 1 to Iter **do** 3:    **for** *k* = 1 to *K* **do** 4:        $v_{k}\left(i+1\right)=w\cdot v_{k}\left(i\right)+c1\cdot Rand_{1}\cdot \left(p_{pbest}\left(i\right)-p\left(i\right)\right)+c2\cdot Rand_{2}\cdot \left(p_{gbest}\left(i\right)-p_{k}\left(i\right)\right)$ 5:        $p_{k}\left(i+1\right)=p_{k}\left(i\right)+v_{k}\left(i+1\right)$ 6:        $IG_{k}\left(i+1\right)=FitnessFun\left(p_{k}\left(i+1\right)\right)$ 7:        **if** $IG_{k}\left(i+1\right)<IG_{pbest}\left(i\right)$ **then**  8:           $p_{pbest}\left(i+1\right)=p_{k}\left(i+1\right)$  9:        **else** 10:           $p_{pbest}\left(i+1\right)=p_{pbest}\left(i\right)$  11:        **end if** 12:        **if** $IG_{pbest}\left(i+1\right)<IG_{gbest}$ **then**  13:           $p_{gbest}\left(i+1\right)=p_{pbest}\left(i+1\right)$  14:        **else** 15:           $p_{gbest}\left(i+1\right)=p_{gbest}\left(i\right)$  16:        **end if** 17:    **end for** 18:    $w=w\cdot w_{damp}$  19:**end for** **Output**:
The best fitness value and its correlated paths as the optimal solutions.

## 4. Numerical Experiments

To evaluate the performance of the proposed RAST* for solving the path planning problem for adaptive sampling, all numerical experiments are carried out in Matlab R2018a under Windows 10 on a computer with Intel(R) Core(TM) i7-6700HQ CPU @ 3.40GHz / 16.0 GB RAM. The proposed RAST* is compared with RRST*, RAST, and standard PSO. The parameters of PSO are set as c1=1, c2=1, w=1, and $w_{damp}=0.98$, population size K=500, maximum number of iterations Iter=100 and number of control points n=5. The sensor range $d_{min}$ is set as 1 km. In particular, the influence of parameters (step size δ, maximum ratio value $P_{r}$) of RAST* is analyzed before performing scenarios.

**Figure 1:**
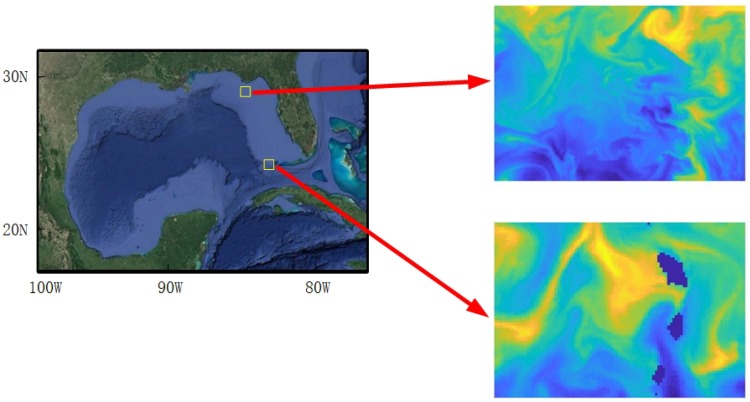
The geographical map for Gulf of Mexico. Two areas are selected for scenario 1 and 2.

The utility map is generated based on sea temperature data from NOAA in the Gulf of Mexico in this research. The website of NOAA provides netCDF files for users to do research. Matlab can decode the netCDF files and draw a figure based on a two dimensional array. Figure 1 shows the geographical map for the Gulf of Mexico, and two areas are selected for scenario 1 and 2.

### 4.1. Preliminary: Parameter Analysis

**Figure 2:**
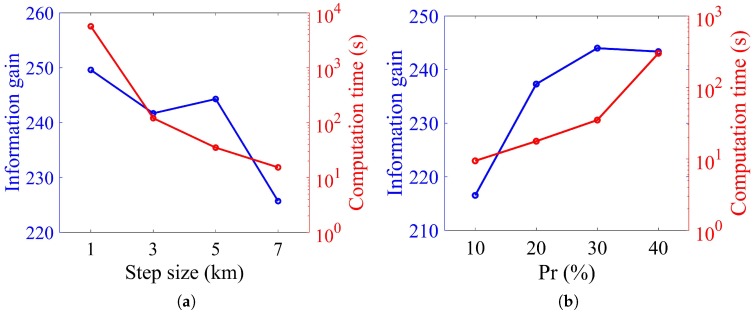
Comparison of parameter analysis for RAST*. (**a**) Step size, (**b**) maximum ratio value.

RAST* has two parameters to adjust that are step size δ and maximum ratio value $P_{r}$, since the best values of these two parameters differ on different problems. To find proper values, statistical parameter analyses are performed over a scientific interest area of 100 km × 70 km for adaptive sampling in a variable ocean environment without obstacles. Computation time and information gathering are applied to evaluate the performance. Results are presented in Figure 2.

Step size sets the distance between the current node and newly generated node. As can be observed from Figure 2a, the larger the step size is, the lower the computation time is, but the less the information gathering is. A step size of 1 km is too computation time consuming when compared to the other three step sizes, although it returns the maximum information gathering. A step size of 7 km is the other way around. A step size of 3 and 5 km have almost the same information gathering, but a step size of 3 km has a higher computation time. Therefore, δ=5 is the proper value for the step size in this research.

The maximum ratio value determines the termination condition. It can be noted from Figure 2b that the larger the ratio value is, the higher the computation time is and the more the information gathering is. Since $P_{r}=30\%$ and $P_{r}=50\%$ have almost the same information gathering and $P_{r}=30\%$ is with less computation time, $P_{r}=30\%$ is the proper value for the maximum ratio value in this research.

### 4.2. Scenario 1: Adaptive Sampling in Variable Ocean Environment without Obstacles

This scenario discusses the performance of RAST*, RRST*, RAST, and PSO over a scientific interest area without obstacles in the Gulf of Mexico on 24 December 2017. The utility map is the normalized sea temperature data of 100×70 grids and each grid represents an area of 1 km × 1 km.

**Figure 3:**
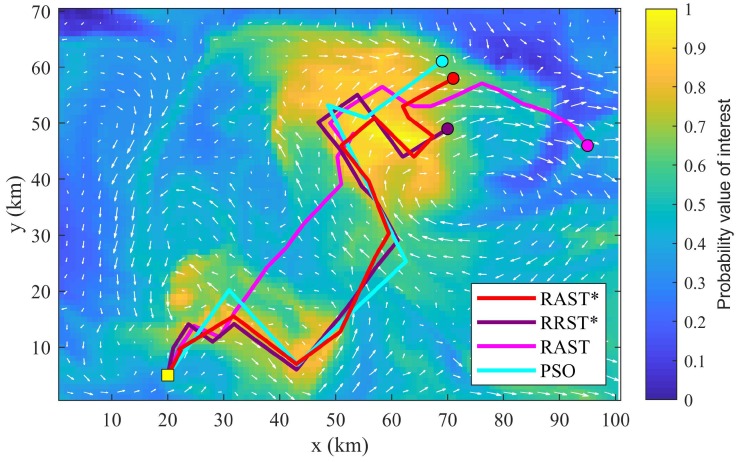
Illustration of optimized paths produced by RAST*, RRST*, RAST, and PSO over a scientific interest area without obstacles. (Scenario 1) The utility map denotes the probability value of scientific interest (blue = low scientific interest, yellow = high scientific interest). White arrows represent variable ocean currents.

Figure 3 displays the results of the optimized paths produced by the four path planners. A UMV, with a constant speed of 2 m/s, starts from (20,5) and follows the planned path to collect information within pre-specified mission time of 50 h. The utility map denotes the probability value of scientific interest (blue = low scientific interest, yellow = high scientific interest), from which we can observe that the proposed RAST* produces the optimal path that explores and covers high scientific interest areas.

Moreover, a 50-run Monte Carlo simulation, which refers to that the numerical simulation is run repeatedly 50 times to check the performance (standard deviation) of each algorithm, is conducted, and results are shown in Table 1. It can be noted that RAST* and RRST* return a similar mean fitness value and standard deviation, but RAST* is computationally faster than RRST*. It is clearly discussed in Section 3.5 that RAST*, RRST*, and RAST have the same time complexity as that of RRT, that is *O*(*n*log*n*), where n denotes the total number of iterations. It means that RAST* can find the optimal solution with less iterations. The main reason for this is that RRST* searches the configuration space with randomness, while RAST* focuses more on high scientific interest areas. This demonstrates the advantage of applying the tournament selection method in the sample phase. RAST, without checking a more "informative” branch, performs poorly in all respects, which reveals that RAST needs significantly more number of iterations to find the optimized solution than that of RAST*. PSO is suboptimal compared to RAST*.

We can conclude that the proposed RAST* is superior to RRST* in terms of computation time, and RAST* is more efficient and effective than RAST and PSO, when performing adaptive sampling in a variable ocean environment without obstacles.

### 4.3. Scenario 2: Adaptive Sampling in Variable Ocean Environment with Obstacles

In this scenario, we examine the performance of RAST*, RRST*, RAST, and PSO over a scientific interest area with obstacles in the Gulf of Mexico on 24 December 2017. The utility map is the normalized sea temperature data of 100×70 grids, and each grid represents an area of 1 km × 1 km.

**Figure 4:**
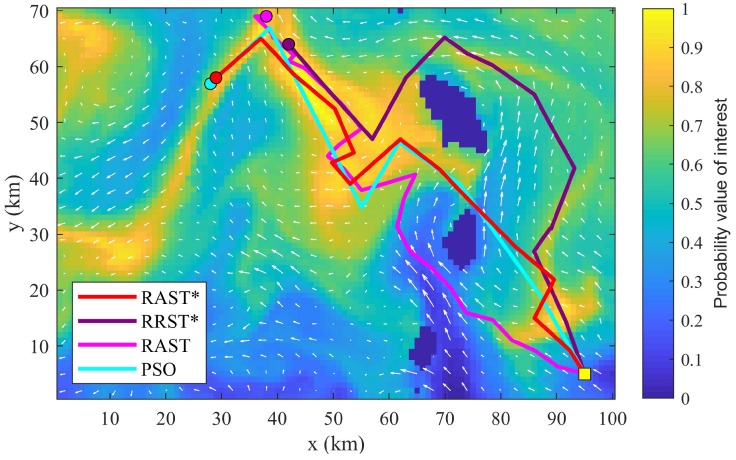
Illustration of optimized paths produced by RAST*, RRST*, RAST, and PSO over a scientific interest area with obstacles. (Scenario 2) The utility map denotes the probability value of scientific interest (blue = low scientific interest, yellow = high scientific interest, darkest blue = obstacles). White arrows represent ocean currents.

Figure 4 shows a comparison of the optimized paths produced by the four path planners in an obstacle environment. A UMV, with a constant speed of 2 m/s, starts from (95,5) and follows the planned path to collect information within a pre-specified mission time of 50 h. As can be noticed, the way passing through obstacles differs on different algorithms. The path of RAST goes downstream and makes good use of ocean currents so as to save traveling time, but the chosen road is with low scientific interest. The path of RRST* makes a detour around obstacles and wastes traveling time on where scientific interest is not high. RAST* and PSO choose the road that will encounter a short period of counter current but leads to high scientific interest areas quickly. Consequently, RAST* and PSO result in gaining more information than RRST* and RAST, as shown in "Maximum IG” in Table 2.

From Table 2, it can be concluded that neither PSO nor RAST can be selected for practical application because the standard deviation of PSO is too large and RAST is computation time consuming. RRST* lacks competitiveness due to low computational efficiency when compared to RAST*.

Among the four path planners, RAST* outperforms RRST*, RAST, and PSO, when performing adaptive sampling in variable ocean environment with obstacles.

### 4.4. Robustness Assessment

To further verify the performance of each algorithm, three different tests with different grid maps are presented to assess the robustness and performance of these algorithms. The first test involves ten randomly selected grid maps of 50×35 grids, and each grid represents an area of 3 km × 3 km. The second test involves ten randomly selected grid maps of 100×70 grids and each grid represents an area of 1 km × 1 km. The third test involves ten randomly selected grid maps of 200×140 grids and each grid represents an area of 0.5 km × 0.5 km. The above three tests were performed with randomly selected areas from NOAA in the Gulf of Mexico on 24 December 2017. The input parameter of each algorithm is the same as those in Section 4.1. The information gathering of each algorithm about the three tests are shown in Table 3, Table 4 and Table 5.

As can be seen from the results in these tables, it is worth noting that in such scenarios, the RAST* still has a significantly higher chance of collecting more ocean data than the other three algorithms within pre-specified mission time. This indicates that the ability of information gathering of RAST* outperforms the RRST*, RAST, and PSO. In summary, these three tests demonstrate the robustness and performance of the proposed RAST*.

## 5. Field Experiments

**Figure 5:**
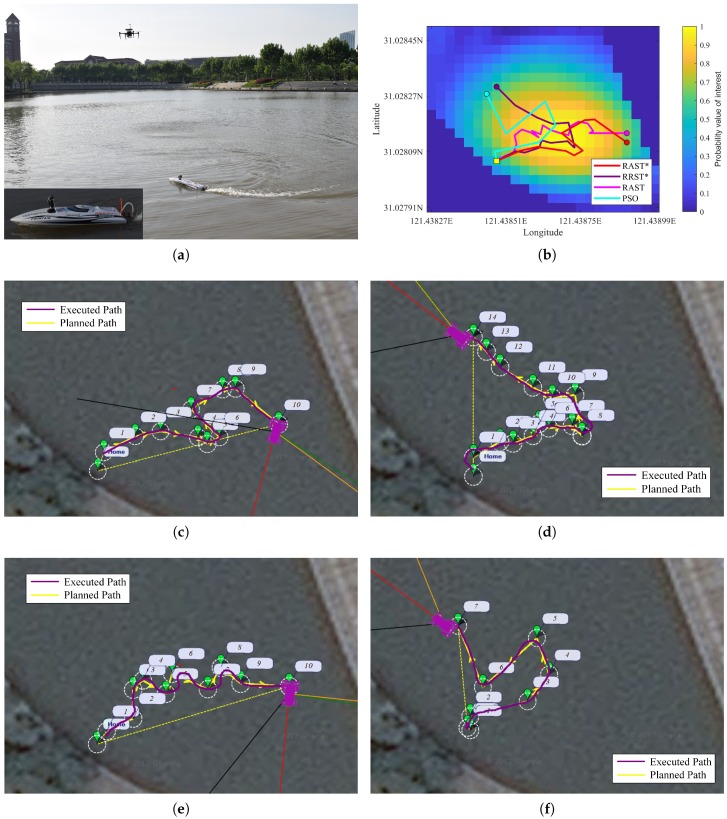
(**a**) Filed experiments of an ASV developed by Shanghai Jiao Tong University. (**b**) Numerical results of off-line paths produced by the four path planners. The background is the simulated utility map of Lake Zhiyuan. (**c**) Interface of the recorded executed path produced by the proposed RAST* path planner in Mission Planner. (**d**–**f**) Interface of the recorded executed path produced by RRST*, RAST, and PSO path planners in Mission Planner.

To assess the superiority and effectiveness of the four path planners, field experiments were performed in Lake Zhiyuan, Shanghai, China. An autonomous surface vehicle (ASV), with easy deployment and real time data transmission, is employed to maximize water sample acquisition over a virtual scientific interest area with a pre-specified mission time. The ASV, shown in Figure 5a, is actuated by one thruster and one rudder at the back, and equipped with a lithium-ion rechargeable battery, an inertial measurement unit (IMU) and real-time kinematic GPS for localization, a pixhawk and wireless data transmission module for remote control, and some measurement devices, such as temperature sensor and depth sensor.

It is assumed that the lake is calm with very little movement of water and the currents of the lake are ignored. The ASV is tasked to travel with the speed of 1.2 m/s and finish the sampling mission in 60 s. The utility map for field experiments is a combination of real obstacles in the Zhiyuan Lake and the NOAA temperature data in a randomly selected region. Numerical experiments are performed to generate the off-line optimal path for field experiments, shown in Figure 5b. During field experiments, the ASV follows discrete waypoints of an optimized trajectory generated by the four path planners. The performance characteristics of ASV are recorded and transmitted back to the ground station through a data transmission module in real time and the Mission Planner software can output the executed trajectory of the ASV, as shown in Figure 5c,d,e,f.

Table 6 records information gathering of ASV along executed paths based on the four path planners in the virtual utility map with the same constant speed of ASV and the same mission time. It can be noted that the ASV, following the optimized path generated by the RAST* path planner, can gather more information than the other three path planners.

Experimental results further demonstrate the superiority and robustness of the proposed RAST*.

## 6. Conclusions

In this research, we presented a novel variant of the sample-based path planning method for adaptive sampling. The proposed RAST* method integrates a tournament selection method, informative heuristic function, and tree structure of RRT* into a unified path planner. This arrangement enhances exploration and coverage in high scientific interest areas while saving computation time. We validate the proposed RAST* path planner through numerical experiments with a variable ocean environment. The numerical results show that RAST* generates a collision-free and near optimal path of the UMV with more information gathering and less computation time while satisfying constraints on pre-specified mission time, when compared to RRST*, RAST, and PSO. Furthermore, results of field experiments demonstrate the superiority and effectiveness of the proposed RAST* path planner.

In the future, we plan to consider more complicated scenarios, such as avoiding dynamic obstacles [36,37], real-time path re-planning [38,39,40] and cooperation of multiple vehicles [41,42]. Another extension of this work is to develop adaptive stepsize [43] in the RAST* algorithm for further saving computation time.

## Figures and Tables

**Table 1:** Performance comparison of rapidly-exploring adaptive sampling tree star (RAST*), rapidly-exploring random sampling tree star (RRST*), rapidly-exploring adaptive sampling tree (RAST), and particle swarm optimization (PSO) for Scenario 1.

Algorithms	Maximum IG	Mean IG	Std	Mean Computation Time (s)
RAST*	244.3	222.0	9.67	35.7
RRST*	247.2	227.5	9.65	115.6
RAST	213.6	192.3	9.65	3463.7
PSO	239.9	212.2	14.01	60.9

**Table 2:** Performance comparison of RAST*, RRST*, RAST, and PSO for Scenario 2.

Algorithms	Maximum IG	Mean IG	Std	Mean Computation Time (s)
RAST*	237.8	220.1	9.83	103.1
RRST*	229.7	220.1	5.45	609.9
RAST	213.3	191.0	8.89	2161.7
PSO	236.6	198.2	12.85	149.4

**Table 3:** Information gathering of RAST*, RRST*, RAST, and PSO over ten randomly selected scientific interest areas with 50×35 grids. The maximum information gathering for each scenario has been highlighted in bold.

Scenario	RAST*	RRST*	RAST	PSO
1	**85.5**	83.7	74.0	83.4
2	**89.7**	85.1	83.5	86.9
3	**76.3**	71.8	70.8	71.1
4	96.6	**97.0**	92.5	96.4
5	**78.6**	75.0	74.8	73.6
6	**81.2**	80.5	75.6	76.5
7	**85.0**	83.6	81.9	81.6
8	**88.1**	85.5	83.2	86.3
9	**83.3**	81.2	78.5	80.9
10	**91.5**	90.3	86.2	89.2

**Table 4:** Information gathering of RAST*, RRST*, RAST, and PSO over ten randomly selected scientific interest areas with 100×70 grids. The maximum information gathering for each scenario has been highlighted in bold.

Scenario	RAST*	RRST*	RAST	PSO
1	153.7	152.1	152.8	**155.4**
2	**224.2**	203.9	182.3	219.6
3	**214.6**	212.5	208.5	198.7
4	**200.3**	178.8	179.7	197.3
5	**218.8**	205.9	199.8	214.7
6	**214.2**	175.8	148.2	200.1
7	165.5	160.7	158.4	**169.5**
8	187.3	**189.8**	165.6	185.9
9	**225.6**	158.9	161.5	219.7
10	**186.4**	185.6	181.8	172.9

**Table 5:** Information gathering of RAST*, RRST*, RAST, and PSO over ten randomly selected scientific interest areas with 200×140 grids. The maximum information gathering for each scenario has been highlighted in bold.

Scenario	RAST*	RRST*	RAST	PSO
1	**409.5**	372.4	405.6	407.8
2	**488.6**	456.0	458.4	454.4
3	**476.7**	432.6	426.6	465.4
4	**409.4**	365.9	335.8	387.6
5	**415.2**	402.3	389.6	359.1
6	389.1	380.4	365.0	**390.5**
7	**495.8**	455.9	468.9	489.2
8	**438.0**	408.9	398.6	388.4
9	491.6	**495.4**	466.1	480.3
10	**377.9**	355.1	341.2	358.6

**Table 6:** Information gathering of the autonomous surface vehicle (ASV) in field experiments.

Algorithms	IG
RAST*	29.03
RRST*	28.68
RAST	23.75
PSO	25.88

## References

[1] Ferri G., Cococcioni M., Alvarez A. (2015). Mission Planning and Decision Support for Underwater Glider Networks: A Sampling on-Demand Approach. Sensors.

[2] Hitz G., Galceran E., Garneau M.È., Pomerleau F., Siegwart R. (2017). Adaptive continuous-space informative path planning for online environmental monitoring. J. Field Robot..

[3] Hernández J., Istenič K., Gracias N., Palomeras N., Campos R., Vidal E., García R., Carreras M. (2016). Autonomous Underwater Navigation and Optical Mapping in Unknown Natural Environments. Sensors.

[4] Khan J., Cho H.S. (2016). Data-Gathering Scheme Using AUVs in Large-Scale Underwater Sensor Networks: A Multihop Approach. Sensors.

[5] Rudnick D.L. (2016). Ocean Research Enabled by Underwater Gliders. Annu. Rev. Mar. Sci..

[6] Liu Z., Zhang Y., Yu X., Yuan C. (2015). Unmanned surface vehicles: An overview of developments and challenges. Annu. Rev. Control.

[7] Chu Z., Xiang X., Zhu D., Luo C., Xie D. (2018). Adaptive Fuzzy Sliding Mode Diving Control for Autonomous Underwater Vehicle with Input Constraint. Int. J. Fuzzy Syst..

[8] Zeng Z., Lian L., Sammut K., He F., Tang Y., Lammas A. (2015). A survey on path planning for persistent autonomy of autonomous underwater vehicles. Ocean Eng..

[9] Ryan N., Ellen C., Beth A., David A., Burton H., Gaurav S. (2010). USC CINAPS Builds bridges: observing and monitoring the southern california. IEEE Robot. Autom. Mag..

[10] Yu C., Xiang X., Wilson P.A., Zhang Q. (2020). Guidance-Error-Based Robust Fuzzy Adaptive Control for Bottom Following of a Flight-Style AUV with Saturated Actuator Dynamics. IEEE Trans. Cybern..

[11] Yazdani A.M., Sammut K., Yakimenko O., Lammas A. (2020). A survey of underwater docking guidance systems. Robot. Auton. Syst..

[12] Lu D., Xiong C., Zeng Z., Lian L. (2019). Adaptive Dynamic Surface Control for a Hybrid Aerial Underwater Vehicle With Parametric Dynamics and Uncertainties. IEEE J. Ocean. Eng..

[13] Zhang Q., Zhang J., Chemori A., Xiang X. (2018). Virtual Submerged Floating Operational System for Robotic Manipulation. Complexity.

[14] Yu C., Xiang X., Lapierre L., Zhang Q. (2018). Robust Magnetic Tracking of Subsea Cable by AUV in the Presence of Sensor Noise and Ocean Currents. IEEE J. Ocean. Eng..

[15] Mahmoud Zadeh S., Powers D.M.W., Sammut K., Yazdani A.M. (2018). A novel versatile architecture for autonomous underwater vehicle’s motion planning and task assignment. Soft Comput..

[16] McMahon J., Plaku E. (2017). Autonomous Data Collection with Limited Time for Underwater Vehicles. IEEE Robot. Autom. Lett..

[17] Ryan N., Monitoring O., Blackwell W., Wiley J., Link C., Smith R.N., Smith S.L. (2014). Persistent ocean monitoring with underwater gliders: Adapting sampling resolution. J. Field Robot..

[18] Yilmaz N., Evangelinos C., Lermusiaux P., Patrikalakis N. (2008). Path Planning of Autonomous Underwater Vehicles for Adaptive Sampling Using Mixed Integer Linear Programming. IEEE J. Ocean. Eng..

[19] Binney J., Sukhatme G.S. Branch and bound for informative path planning. Proceedings of the 2012 IEEE International Conference on Robotics and Automation.

[20] Hollinger G.A., Sukhatme G.S. (2014). Sampling-based robotic information gathering algorithms. Int. J. Robot. Res..

[21] Ma K.C., Liu L., Heidarsson H.K., Sukhatme G.S. (2017). Data-Driven Learning and Planning for Environmental Sampling. J. Field Robot..

[22] Ma K.C., Liu L., Sukhatme G.S. An information-driven and disturbance-aware planning method for long-term ocean monitoring. Proceedings of the 2016 IEEE/RSJ International Conference on Intelligent Robots and Systems (IROS).

[23] Zhou H., Zeng Z., Lian L. (2017). Adaptive Re-planning of AUVs for Environmental Sampling Missions: A Fuzzy Decision Support System Based on Multi-objective Particle Swarm Optimization. Int. J. Fuzzy Syst..

[24] Lavalle S.M. Rapidly-Exploring Random Trees: A New Tool for Path Planning. http://msl.cs.uiuc.edu/~lavalle/papers/Lav98c.pdf.

[25] Kavraki L.E., Švestka P., Latombe J.C., Overmars M.H. (1996). Probabilistic roadmaps for path planning in high-dimensional configuration spaces. IEEE Trans. Robot. Autom..

[26] Karaman S., Frazzoli E. (2010). Sampling-based Algorithms for Optimal Motion Planning. Int. J. Robot. Res..

[27] Viseras A., Shutin D., Merino L. (2019). Robotic Active Information Gathering for Spatial Field Reconstruction with Rapidly-Exploring Random Trees and Online Learning of Gaussian Processes. Sensors.

[28] Carreras M., Hernandez J.D., Vidal E., Palomeras N., Ribas D., Ridao P. (2018). Sparus II AUV—A Hovering Vehicle for Seabed Inspection. IEEE J. Ocean. Eng..

[29] Wei K., Ren B. (2018). A Method on Dynamic Path Planning for Robotic Manipulator Autonomous Obstacle Avoidance Based on an Improved RRT Algorithm. Sensors.

[30] Cho K., Suh J., Tomlin C.J., Oh S. (2017). Cost-Aware Path Planning under Co-Safe Temporal Logic Specifications. IEEE Robot. Autom. Lett..

[31] Elbanhawi M., Simic M. (2014). Sampling-Based Robot Motion Planning: A Review. IEEE Access.

[32] Arzamendia M., Gregor D., Reina D.G., Toral S.L. (2017). An evolutionary approach to constrained path planning of an autonomous surface vehicle for maximizing the covered area of Ypacarai Lake. Soft Comput..

[33] Kennedy J., Eberhart R. Particle swarm optimization. Proceedings of the International Conference on Neural Networks (ICNN’95).

[34] Yan Z., Li J., Wu Y., Zhang G. (2018). A Real-Time Path Planning Algorithm for AUV in Unknown Underwater Environment Based on Combining PSO and Waypoint Guidance. Sensors.

[35] Zeng Z., Sammut K., Lian L., He F., Lammas A., Tang Y. (2016). A comparison of optimization techniques for AUV path planning in environments with ocean currents. Robot. Auton. Syst..

[36] Zeng Z., Lammas A., Sammut K., He F., Tang Y. (2014). Shell space decomposition based path planning for AUVs operating in a variable environment. Ocean Eng..

[37] Chiang H.T.L., Tapia L. (2018). COLREG-RRT: An RRT-Based COLREGS-Compliant Motion Planner for Surface Vehicle Navigation. IEEE Robot. Autom. Lett..

[38] MahmoudZadeh S., Yazdani A., Sammut K., Powers D. (2018). Online path planning for AUV rendezvous in dynamic cluttered undersea environment using evolutionary algorithms. Appl. Soft Comput..

[39] Yazdani A.M., Sammut K., Yakimenko O.A., Lammas A., Tang Y., Mahmoud Zadeh S. (2017). IDVD-based trajectory generator for autonomous underwater docking operations. Robot. Auton. Syst..

[40] Zeng Z., Sammut K., Lammas A., He F., Tang Y. (2015). Efficient Path Re-planning for AUVs Operating in Spatiotemporal Currents. J. Intell. Robot. Syst. Theory Appl..

[41] Xiong C., Chen D., Lu D., Zeng Z., Lian L. (2019). Path planning of multiple autonomous marine vehicles for adaptive sampling using Voronoi-based ant colony optimization. Robot. Auton. Syst..

[42] Zeng Z., Sammut K., Lian L., Lammas A., He F., Tang Y. (2018). Rendezvous Path Planning for Multiple Autonomous Marine Vehicles. IEEE J. Ocean. Eng..

[43] An B., Kim J., Park F.C. (2018). An Adaptive Stepsize RRT Planning Algorithm for Open-Chain Robots. IEEE Robot. Autom. Lett..

